# The use of virtual reality and augmented reality to enhance cardio-pulmonary resuscitation: a scoping review

**DOI:** 10.1186/s41077-021-00158-0

**Published:** 2021-04-12

**Authors:** Katherine Kuyt, Sang-Hee Park, Todd P. Chang, Timothy Jung, Ralph MacKinnon

**Affiliations:** 1 Manchester University NHS Foundation Trust, Manchester, UK; 2 Korea Institute of Civil Engineering and Building Technology, Seoul, South Korea; 3 Children’s Hospital Los Angeles, Los Angeles, USA; 4 Manchester Metropolitan University, Manchester, UK

**Keywords:** Virtual reality, Augmented reality, Cardiopulmonary resuscitation, Simulation

## Abstract

**Background and objective:**

Virtual reality (VR) and augmented reality (AR) have been proposed as novel methods to enhance cardio-pulmonary resuscitation (CPR) performance and increase engagement with CPR training. A scoping review was conducted to map the global evolution of these new approaches to CPR training, to assess their efficacy and determine future directions to meet gaps in current knowledge.

**Methods:**

A standardised five-stage scoping methodology was used to (1) identify the research question, (2) identify relevant studies, (3) select the studies, (4) chart the data and (5) summarise the findings. The Kirkpatrick model levels of evidence were used to chart and assess the efficacy of each intervention reported. A multi-pronged search term strategy was used to search the Web of Science, PubMed, CINAHL and EMBASE databases up to June 2020.

**Results:**

A total of 42 articles were included in this review. The first relevant paper identified was published in 2009 and based on VR, from 2014 onwards there was a large increase in the volume of work being published regarding VR and AR uses in CPR training. This review reports Kirkpatrick level one to three evidence for the use of VR/AR–CPR. Inconsistencies in the specific language, keywords used and methodologies are highlighted.

**Conclusion:**

VR and AR technologies have shown great potential in the area of CPR, and there is continuing evidence of new novel applications and concepts. As VR/AR research into CPR reaches an inflection point, it is key to bring collaboration and consistency to the wider research community, to enable the growth of the area and ease of access to the wider medical community.

**Supplementary Information:**

The online version contains supplementary material available at 10.1186/s41077-021-00158-0.

## Background

As a society, we currently face two significant problems with cardiopulmonary resuscitation (CPR). The first challenge centres on the quality of CPR performed during both out-of-hospital and in-hospital cardiac arrests. The second challenge lies in trying to engage the general public in CPR training. Effective CPR, with appropriate rate and depth of compressions, is vital for survival after cardiac arrest [1]. Even within hospitals, high-quality chest compressions do not occur in 36–87% of CPRs [2–4]. Studies of CPR training within hospitals, typically face to face training in groups on CPR manikins, indicate substantial room for improvement in CPR performance [5]. Outside of hospitals, bystanders witness approximately 50% of cardiac arrests [6]. It is estimated that neurologically intact survival would increase up to four-fold, saving more than 100,000 deaths a year if the general public was educated and engaged to do CPR [6, 7]. Therefore, providing up to date CPR training, that teaches high-quality skills, in an engaging manner for both healthcare professionals and members of the public, is of great importance. Further to this, the recent novel coronavirus (COVID-19) pandemic has led to an increased urgency to reduce group trainings, especially where trainees are in continued close contact, such as many of the current CPR training methods.

Virtual reality (VR) has been proposed as potentially a powerful tool to enhance interaction and performance with manikin simulators [8]. Augmented reality (AR) solutions to improve CPR engagement, using commercially available devices such as Google Glasses (Google, Mountain View, California, USA) and Microsoft HoloLens (Microsoft, Redmond, Washington, USA), are also appearing [9, 10].

For the purposes of this review, a training device had to provide an immersive experience to be classed as using virtual reality techniques. This is in agreement with Cant et al [11] who suggest a definition of VR within healthcare as a technology that uses fidelity, immersion and patient depiction. Augmented reality should also use technology to provide a multi-sensory experience environment for the user; however, this is not always exclusively immersive. The field of VR and AR based CPR solutions is developing promisingly at the time of this writing, with new proposals and early validation studies. The spread of COVID-19 has led to an increase in interest in how innovative technologies, such as VR and AR, may be utilised to limit the unnecessary physical interaction of people. To date, there has been no review that has focused on the evolution of the VR and AR based CPR solutions. This scoping review aims to review published material on VR and AR in CPR training to evaluate the evidence of its efficacy in enhancing learning, knowledge, skills, and attitudes. Also, to provide suggestions regarding how the field can continue to develop and provide increasing utility in teaching CPR skills.

## Methods

A scoping review provides an initial insight into the content and breadth of developing heterogeneous data from diverse sources, prior to embarking on a systematic review on deeper established datasets [12, 13]. This scoping review utilised the Preferred Reporting Items for Systematic reviews and Meta-Analyses extensions for Scoping Reviews [14]. The methods of this review were based on the five-stage methodology of Levac, Colquhoun and O’Brien [15].

1. Identify the research question
2. Identify relevant studies
3. Select the relevant studies
4. Chart the data
5. Summarise and report the results

### Identify the research question

As technologies including VR and AR continue to evolve, become less expensive and within the reach of researchers, the next step to potential applications in healthcare staff training comes in to sight. Globally, CPR training inside and outside of healthcare remains challenging and the evidence base for the application of VR and AR to CPR is beginning to grow. This growth has led to the proposal of the question ‘Can VR and/or AR improve CPR training?’ This in turn was developed further to focus upon the educational outcomes of basic life support training, using the Kirkpatrick model. The resultant research question of ‘Can VR and/or AR enhance learning, knowledge, skills or attitudes and if applied, was there an outcome concerning basic life support skills?’ This question was deemed broad enough to capture the current evidence base yet focused enough to indicate areas and direction for development of the literature further.

### Eligibility criteria

In order to be included, the identified article had to be on the topic of life support training or performance and use of VR and/or AR as a teaching or feedback tool. No limitations on date or language were filtered for this search; English-language translations were procured for those written primarily in a different language.

Articles were excluded if they were opinion pieces rather than unbiased reviews/studies, or did not use VR or AR methods for CPR. To be classed as virtual reality, the methodology had to include an immersive element.

### Identify relevant studies

A multi-pronged search term strategy was used to search the Web of Science, PubMed, CINAHL and EMBASE databases in June 2020. Articles were required to have terms from both topics of CPR and VR/AR. Terms for CPR included *heart massage*, *artificial respiration*, *cardiopulmonary resuscitation*, *CPR*, *basic life support*, *heart stoppage*, *cardiac massage*, *heart massage*, *circulatory arrest*. VR/AR terms included *computer simulation*, *user-computer interface*, *virtual reality*, *augmented reality*, *computer simulation*, *computer assisted therapy* (Table 1). Google scholar was also searched, as were hand searches on full text articles. Duplicates were removed by an author (KK).

**Table 1:** Full search strategy used for database searches on Web of Science, PubMed, CINAHL and EMBASE

(“heart massage” or “artificial respiration” or “cardiopulmonary resuscitation” or CPR or “basic life support” or “heart stoppage” or “cardiac massage” or “heart massage” or “circulatory arrest”)	
And	
(“computer simulation” or “user-computer interface” or “virtual reality” or “augmented reality” or “computer simulation” or “computer assisted therapy”)	

### Study selection

One author and an expert librarian conducted the search. Three authors screened and full text reviewed the resultant findings (KK, S-HP and RM); study selection was confirmed by discussion and consensus by all the authors.

### Charting the data

Microsoft Excel 2013 (Redmond, WA, USA) was used to gather and display relevant information regarding each of the selected studies. The data-charting matrix was developed collaboratively by two authors (RM and TC) to determine which metrics and data to collect. This included subject of study (virtual reality or augmented reality), year of publication, geography of study, source of publication and impact factor (June 2020).

The findings of the studies were assessed and reported based on the Kirkpatrick model, which is used to assess the efficacy of a specified training or intervention. The Kirkpatrick model can be used to evaluate how likely a training programme is to meet the needs of both the organisation and the trainees. It consists of four levels of evidence, assessing the reaction to training, skills learnt, behaviours and outcomes [16] (Table 2).

**Table 2:** The Kirkpatrick levels of evidence to assess intervention efficacy

Level 1	Participants react favourably to the learning or intervention.
Level 2	Participants acquired knowledge, skills and attitudes based on the intervention or study.
Level 3	Participants applied what they learnt into practice.
Level 4	Once applied, there was an outcome to that application of skills learnt from the intervention.

### Synthesis and reporting of results

Studies were grouped according to the main method used/reported, that of VR or AR. Also, studies were stratified according to the Kirkpatrick model, and which level of evidence they supplied. The information collated in this literature search was reviewed to provide an overview of the existing knowledge and findings on this topic and identify gaps in the literature.

## Results

**Fig. 1:**
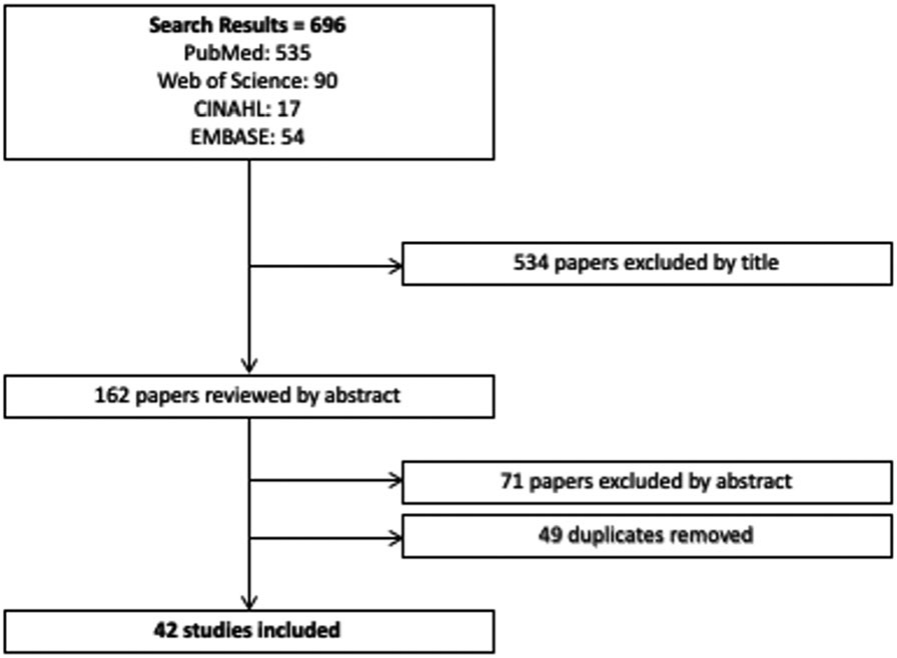
Flow diagram of the review of search results for the literature review

The search resulted in 696 articles, of which 534 were excluded by title, and 71 were excluded on review of the abstract. An additional 49 studies were identified as duplicates and removed. Forty-two articles were included in this review (Fig. 1). Of these articles 29 were regarding VR, and 13 were studies based on AR. A full list of included articles can be found in Additional file 1.

### Publications by year

**Fig. 2:**
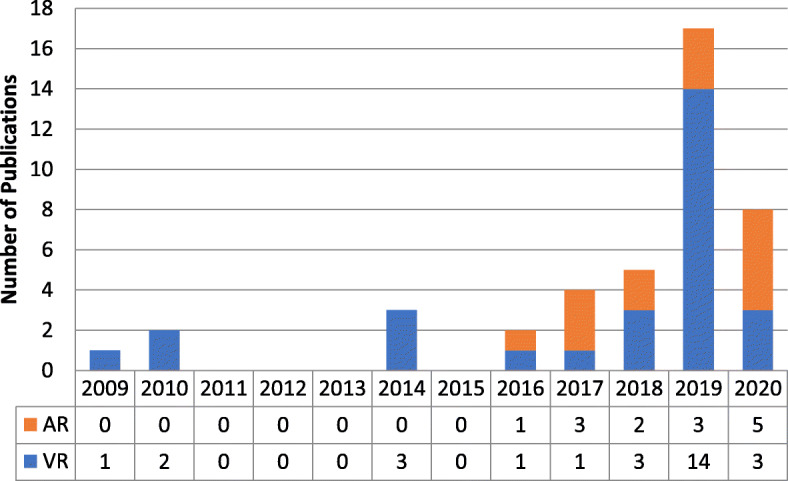
Graphical representation of publications per year. Data collected June 2020

The first original article published on these topics was in 2009 and reported on the acceptance of, and interest in, a newly developed prototype of a virtual reality enhanced manikin (VREM) [17]. The following year, further data on this VREM was presented during a conference [18]. In addition, in 2010, a review discussing the potential uses of VR in nursing education and CPR was published [19]. From 2016, there has been an exponential growth of publications regarding VR and AR in CPR. Although the data was collected only part way through 2020, the expectation is for this trend to continue. Earlier publications reviewed the use of VR; the first of the included publications based on the use of AR technology was not published until 2016 [20] (Fig. 2).

### Geography of publications

Publications were identified from twelve different countries (see Table 3). The first original article on these topics was published by an Italian team in 2009 [17] and this team has since continued to publish on the topic [18, 21–23]. Research groups in the USA have also significantly contributed to the literature on the use of VR and AR in CPR. This review identified 16 publications (6 articles, and 10 conference abstracts) from primarily USA based teams ranging in publication from 2010 to 2020. In contrast, although British teams have published multiple papers on the subject [24–27], the first of these papers was not published until 2019.

**Table 3:** Nationality of the research teams and year of first publication from that country. When authors were from multiple nations, the nationality of first author was used

Country	Year of first publication
Canada	2019
Germany	2018
Italy	2009
Japan	2017
Netherlands	2020
Singapore	2018
Spain	2017
Sri Lanka	2019
Switzerland	2016
Turkey	2019
UK	2019
USA	2010

### Source of publication

Twenty-four publications were articles, and 18 were published abstracts from conferences. A number of the articles were published in high impact journals such as Circulation. Resuscitation, another journal with a high impact factor, has published many publications on the topics of VR and AR in CPR; both conference abstracts and full articles (Additional file 2).

### Author keywords

Authors used a large variety of keywords throughout the papers. The most prominent words were ‘reality’, ‘resuscitation’, ‘virtual’. There was a discrepancy in the use of ‘VR’ versus ‘virtual reality’. Additionally, some excluded papers had used the term ‘virtual reality’, where the study protocol did not align with standard definitions of VR as of this publication.

### Assessment of outcomes—Kirkpatrick model

#### Level 1: feasibility and reaction

Regardless of the effectiveness of VR and AR in CPR training and provision, they are of limited utility unless they are accepted by the workforce and trainers. This aligns with level one of evidence in the Kirkpatrick model. In the first novel paper included in this review, Semeraro et al (2009) evaluated the acceptance of the VREM (VR enhanced manikin) prototype, 85% of participants stated the VR experience was interesting and believed it could be very useful for healthcare training [17]. However, the sample for this study consisted of volunteers to review the manikin who were already attending a medical congress, and therefore have a large potential to be biased.

Wong et al (2018) surveyed 30 CPR instructors regarding their views on the use of VR in CPR training, reporting that VR was viewed by the instructors as having potential as a blended learning tool, for both novice and experienced healthcare professionals [28]. Additionally, there has been positive feedback from participants who used VR for CPR training, with reports of an increase in skill confidence [25, 29], and users finding VR user friendly [10, 17, 30]. Recently, Balian et al. have expanded the concept of AR CPR training further. Participants undergoing the AR training (*n* = 51) had real-time feedback, via a holographic overlay of blood flow to vital organs, which was dependent on the quality of chest compressions being performed. Ninety-eight percent of the participants felt the visualizations were helpful for training, and 94% were willing to use the application in future CPR training [31].

#### Level 2: knowledge, skill and attitudes

The second level of evidence in the Kirkpatrick model is evidence of participants learning new knowledge or skills. Multiple studies found that the use of VR and AR during CPR training led to an increased in the practical skills of CPR [25, 29, 32]. The skill increase post VR/AR-based trainings were shown to be at least comparable with traditional classroom methods; however, it is not established that VR and AR leads to greater skill improvement [30, 33, 34]. Siebert et al (2017) studied the effects of wearing AR glasses during a simulated paediatric arrest. They compared the adherence to American Heart Association guidelines when wearing AR glasses or when referring to pocket reference cards. Although many measured outcomes did not change, participants wearing the glasses demonstrated improved adherence with administrating the correct defibrillation doses [35].

#### Level 3: application of knowledge

Multiple studies [36, 37] went beyond looking at purely the skills of CPR and used VR and AR technology to immerse participants into a simulated scenario in which they would be required to use their CPR skills. These scenarios attempt to address level three of the Kirkpatrick model, the application of knowledge. Studies showed increased engagement with the scenarios during VR and AR-based training [36, 37]. Furthermore, Perez et al (2017) concluded that physician feedback via google glasses during a simulated cardiac arrest improved the rate of successful CPR [38].

#### Level 4: outcomes

The highest level of evidence for a teaching/intervention using the Kirkpatrick model is an improvement in patient/cardiac arrest victim outcomes. The authors were not able to identify any published work on the influence of VR and AR training on patient outcomes.

## Discussion

The aim of the study was to evaluate the development of research into the use of VR and AR to enhance CPR training and provision. The findings of this review indicate that this field of VR and AR CPR is both diverse and immature. There is also evidence that the field is growing and evolving with increasing publications and subsequent citations stimulating the growth. Geographically, there is a spread of activity across developed countries, as expected of a new field that is highly dependent on advances in technology. The high number of novel innovations and experimental studies presented indicate that the field is ripe for future research and development. The higher proportion of VR studies may be reflective of the state of current technology. It is expected that the number of research articles regarding the use of AR in CPR may increase in coming years.

Several of the papers identified in this search were abstracts or details of innovative designs where the testing and results had not yet been published. This illustrates the growth and potential of this area of study, and reflects the development and incorporation of high fidelity manikins into emergency medicine [39]. Innovative designs varied from custom-designed VR wearable devices [40], to encouraging engineering students to engage with CPR training by designing a smartphone app [41]. The majority of papers identified focused on CPR skills in healthcare professionals, which aligns with the introduction of novel technology as it allows comparison to the inbuilt control of previous performance. However, several studies did recruit laypeople [29, 36]. Additionally, novel work by Lopez-Belmonte et al. demonstrated that AR methods could be used to teach the basics of CPR compressions to children as young as 5 years old [32].

The reviewed publications show a wide range of methodologies to assess the use of VR and AR. Overall, the studies illustrated that virtual technologies have been well received by instructors and those undergoing training (healthcare professionals and laypeople). There is evidence to show that VR and AR technologies are particularly well received by the ‘technology natives’ of the younger generation [29, 32]. By engaging school children in learning CPR skills, we are enabling them to enter into adulthood with the skills to improve the rates of bystander intervention. As AR develops, the potential uses of being able to augment the users physical surrounding with the virtual world are numerous. The work of Balian et al. is an example of this; by adding the component of the user seeing the blood circulation around the manikin, there is the added aspect of not only auditory feedback but also visual [31]. AR expands on the technology of VR and is a more recent development, therefore there are less papers regarding the use of AR and CPR. Javaheri et al. developed an AR CPR trainer model, where participants can learn and practice CPR skills out of a classroom setting, using AR via a virtual teacher that instructs and provided feedback [42]. Similar work has also been presented by Moe et al. [43].

The high rigor of the science generated to date is reflected by the number of publications in high impact peer reviewed journals. This may also indicate that the novelty of VR is of interest as of this writing, to editors and readerships. Reviewing the authorship of the included publications, it can be seen that there are emerging research groups; where the same team have several publications in the field of VR/AR CPR based in both Italy and the USA.

A lack of systematic reporting in terms of author keywords and lack of agreed-upon terminology was identified, which has led to a lack of cohesion in how work is categorised within repositories. Of note, it was apparent that there is a lack of consensus of what constitutes VR. Does virtual reality have to be a fully immersive experience, or can you class any situational-based learning using technology as VR? Kardong-edgren et al [44] discussed the issue of the differing definitions of VR and concluded that the key aspect was immersion into an environment through sensory richness. The same discrepancies are likely to occur as research into AR, which at this stage should be used only to describe the live overlay of virtual situations on top the subject’s physical surroundings. However, as AR builds on rather than replaces the user’s surroundings, this could be through a screen such as a tablet or smart phone, or through an immersive headset.

These factors are likely to be limiting the visibility of VR and AR research, both internally within the simulation medicine community, and externally to the wider reader and clinician. Standardization of keywords and terminology has been used in simulation-based education [29] and would likely be on benefit in VR and AR, providing an opportunity to coalesce researchers and advance the field further.

Currently, the field of AR/VR CPR research is likely to be at an inflection point in terms of publications as researchers within this topic span both experts in the digital innovation and resuscitation science and the technology continues to rapidly advance. However, to enable successful growth, it is vital to collaborate as a community of practice that mutually engages, shares resources and has joint accountability to shape this field of research and yield potential solutions to improve CPR quality and bystander CPR engagement. The International Network for Simulation-based Pediatric Research, Innovation & Education (INSPIRE network) is an example of where this has been achieved [45]. It was established to provide a framework of research needs and standardisation within paediatric simulation-based science and medicine [36] and encourages collaboration between persons and institutions [45]. Another forward step would be an agreement of a lexicon of terms and definitions to clarify the field and aid appropriate signposting for research findings and resources.

The COVID-19 pandemic has led to a ‘new-normal’ that poses significant challenges on how to learn and maintain CPR skills. Gatherings of large groups are strongly discouraged and, in many organisations, not allowed at all, the majority learning has been converted to a socially distant or virtual format, and people are encouraged to keep a distance of 2-metres or more unless wearing suitable persona protective equipment. Further collaborative exploration of the value of VR and AR CPR may provide important insights on potential solutions to these challenges, and the changing landscape of CPR training.

### Limitations

The analytical methodology chosen was not a systematic review, therefore the quality of the papers was not reviewed in-depth, and all were treated with equal weighting. Furthermore, our search terms selected may have missed other unique articles that could have otherwise met inclusion criteria, as the keywords for new technologies may still be evolving.

## Conclusion

From 2009 to 2019, the number of peer-reviewed articles and conference proceedings about VR and AR on CPR has grown slowly but now exponentially, with geographical diversity and high impact. Published work has demonstrated that VR and AR technologies met level one and two in the Kirkpatrick model to assess a teaching method. Early evidence suggests that trainees apply the knowledge they learn during VR and AR training sessions, but further evidence is needed, along with data regarding the outcome of the application of skills. Now is the time to look to the future, building on the work reviewed in this paper, and grasping the opportunity for new innovations to improve how healthcare professionals and the general public provide CPR.

## Supplementary Information

**Additional file 1.** The studies included in the scoping review.

**Additional file 2.** The impact factor of the journals publishing VR AR CPR studies, in this review.

## Data Availability

We did not collect any data or materials in this research.

## References

[1] Wallace SK, Abella BS, Becker LB (2013). Quantifying the effect of cardiopulmonary resuscitation quality on cardiac arrest outcome: a systematic review and meta-analysis. Circ Cardiovasc Qual Outcomes.

[2] Sutton RM, Niles D, Nysaether J, Abella BS, Arbogast KB, Nishisaki A (2009). Quantitative analysis of CPR quality during in-hospital resuscitation of older children and adolescents. Pediatrics..

[3] Sutton RM, Wolfe H, Nishisaki A, Leffelman J, Niles D, Meaney PA (2013). Pushing harder, pushing faster, minimizing interruptions… but falling short of 2010 cardiopulmonary resuscitation targets during in-hospital pediatric and adolescent resuscitation. Resuscitation..

[4] Cheng A, Brown LL, Duff JP, Davidson J, Overly F, Tofil NM (2015). Improving cardiopulmonary resuscitation with a CPR feedback device and refresher simulations (CPR CARES Study): a randomized clinical trial. JAMA Pediatr.

[5] Taniguchi D, Baernstein A, Nichol G (2012). Cardiac arrest: a public health perspective. Emerg Med Clin North Am.

[6] London Ambulance Service NHS Trust Cardiac Arrest Annual Report 2018/19 (2019). Clinical Audit and Research Unit, London Ambulance Service NHS Trust.

[7] Bohn A, Van Aken H, Lukas RP, Weber T, Breckwoldt J (2013). Schoolchildren as lifesavers in Europe—training in cardiopulmonary resuscitation for children. Best Pract Res Clin Anaesthesiol.

[8] Haans A, Ijsselsteijn W (2006). Mediated social touch: a review of current research and future directions. Virtual Reality.

[9] Chaballout B, Molloy M, Vaughn J, Brisson Iii R, Shaw R (2016). Feasibility of augmented reality in clinical simulations: using google glass with manikins. JMIR Med Educ.

[10] Ingrassia PL, Carfagna F, Mormando G, Giudici E, Strada F, Lamberti F, et al. Augmented reality learning environment for basic life support and defibrillation training: usability study. J Med Internet Res. 2020;22(5):e14910. 10.2196/14910 PMC7251481 32396128

[11] Cant R, Cooper S, Sussex R, Bogossian F. What's in a Name? Clarifying the Nomenclature of Virtual Simulation. Clinical Simulation in Nursing. 2019;27:26–30.

[12] Whittemore R, Knafl K (2005). The integrative review: updated methodology. J Adv Nurs.

[13] Williams B, Reddy P, Marshall S, Beovich B, McKarney L (2017). Simulation and mental health outcomes: a scoping review. Adv Simul.

[14] Tricco AC, Lillie E, Zarin W, O'Brien KK, Colquhoun H, Levac D (2018). PRISMA extension for scoping reviews (PRISMA-ScR): checklist and explanation. Ann Intern Med.

[15] Levac D, Colquhoun H, O'Brien KK (2010). Scoping studies: advancing the methodology. Implement Sci.

[16] Bates R (2004). A critical analysis of evaluation practice: the Kirkpatrick model and the principle of beneficence. Eval Prog Plan.

[17] Semeraro F, Cerchiari EL, Frisoli A, Bergamasco M (2009). Virtual reality enhanced mannequin (VREM) that is well received by resuscitation experts. Resuscitation..

[18] Semeraro F, Frisoli A, Bergamasco M, Cerchiari EL. Mini VREM project (mini virtual reality enhanced mannequin). Resuscitation. 2010;81:S106. 10.1016/j.resuscitation.2008.12.016 19203823

[19] Kilmon CA, Brown L, Ghosh S, Mikitiuk A (2010). Immersive virtual reality simulations in nursing education. Nurs Educ Perspect.

[20] Siebert JN, Gervaix A, Haddad K, Lacroix L, Manzano S, Ehrler F, et al. Adherence to AHA Guidelines When adapted for augmented reality glasses for assisted pediatric cardiopulmonary resuscitation: a randomized controlled trial. J Med Internet Res. 2017;19(5). 10.2196/jmir.7379 PMC5468544 28554878

[21] Semeraro F, Scapigliati A, Ristagno G, Luciani A, Gandolfi S, Lockey A, et al. Virtual Reality for CPR training: how cool is that? Dedicated to the "next generation". Resuscitation. 2017;121. 10.1016/j.resuscitation.2017.09.024 28951295

[22] Semeraro F, Ristagno G, Grieco N, Scelsi S, Boccuzzi A, Di Marco S, et al. Virtual Reality CPR: A new way to learn CPR. Resuscitation. 2018;130.

[23] Semeraro F, Ristagno G, Giulini G, Gnudi T, Kayal JS, Monesi A (2019). Virtual reality cardiopulmonary resuscitation (CPR): Comparison with a standard CPR training mannequin. Resuscitation.

[24] Vaughan N, John N, Rees N (2019). CPR Virtual Reality Training Simulator for Schools. 2019 International Conference on Cyberworlds (CW).

[25] Bench S, Winter C, Francis G. Use of a virtual reality device for basic life support training: prototype testing and an exploration of users' views and experience. Simul Healthc. 2019;14(5):287–92. 10.1097/SIH.0000000000000387 31490865

[26] Rushton DIA, Campion SP, O'Hare JJ (2020). The use of immersive and virtual reality technologies to enable nursing students to experience scenario-based, basic life support training—exploring the impact on confidence and skills. Comput Inform Nurs.

[27] Gillespie R, Nicholson J, Bickerdike S, Frith G, Hassan T. Comparison of virtual reality versus standard video for point-of-training feedback after cardiopulmonary resuscitation simulation: a mixed methods study. Resuscitation. 2019;142:e58.

[28] Wong MAME, Zary N, Chue S, Jong M, Benny HWK. Clinical instructors' perceptions of virtual reality in health professionals' cardiopulmonary resuscitation education. SAGE Open Med. 2018;6:2050312118799602. 10.1177/2050312118799602 PMC6144504 30245815

[29] Gent L, Sarno D, Coppock K, Axelrod DM. Successful virtual reality cardiopulmonary resuscitation training in schools: Digitally linking a physical manikin to a virtual lifesaving scenario. Circulation. 2020;140(Suppl_2):A396.

[30] McGovern SK, Balian S, Bhardwaj A, Abella B, Blewer AL, Leary M. A Comparison of CPR quality using an augmented reality application versus a standard audio-visual feedback manikin. Circulation. 2020;140(Suppl_2):A455. 10.3389/fdgth.2020.00001 PMC8521903 34713015

[31] Balian S, McGovern S, Abella B, Blewer A, Leary M. Feasibility of an augmented reality cardiopulmonary resuscitation training system for health care providers. Heliyon. 2019;5:e02205. 10.1016/j.heliyon.2019.e02205 PMC6684477 31406943

[32] López Belmonte J, Pozo Sánchez S, López BG. The effectiveness of augmented reality in children's classrooms: a study of learning from SVB and CPR in 5-year-old students. Pixel-Bit. 2019;55:157–78.

[33] Leary M, Almodovar A, Buckler DG, Blewer AL, Abella BS. Using immersive virtual reality to observe differences in lay provider response to an unannounced simulated sudden cardiac arrest based on demographics. Circulation. 2017;136. 10.1097/SIH.0000000000000338 30407959

[34] Khanal P, Vankipuram A, Ashby A, Vankipuram M, Gupta A, Drumm-Gurnee D (2014). Collaborative virtual reality based advanced cardiac life support training simulator using virtual reality principles. J Biomed Inform.

[35] Siebert JN, Ehrler F, Gervaix A, Haddad K, Lacroix L, Schrurs P (2017). Adherence to AHA guidelines when adapted for augmented reality glasses for assisted pediatric cardiopulmonary resuscitation: a randomized controlled trial. J Med Internet Res.

[36] Leary M, Almodovar A, Buckler DG, Bhardwaj A, Blewer AL, Abella BS (2019). Using an immersive virtual reality system to assess lay provider response to an unannounced simulated sudden cardiac arrest in the out-of-hospital setting. Simul Healthc.

[37] Leary M, McGovern SK, Chaudhary Z, Patel J, Abella BS, Blewer AL (2019). Comparing bystander response to a sudden cardiac arrest using a virtual reality CPR training mobile app versus a standard CPR training mobile app. Resuscitation.

[38] Pérez Alonso N, Pardo Rios M, Juguera Rodriguez L, Vera Catalan T, Segura Melgarejo F, Lopez Ayuso B (2017). Randomised clinical simulation designed to evaluate the effect of telemedicine using Google Glass on cardiopulmonary resuscitation (CPR). Emerg Med J.

[39] McFetrich J (2006). A structured literature review on the use of high fidelity patient simulators for teaching in emergency medicine. Emerg Med J.

[40] Buckler DG, Alfredo A, Abella BS, Leary M, Snobelen P, Blewer A (2019). Observing the stages of bystander intervention in virtual reality simulation. World J Emerg Med World J Emerg Med.

[41] Ohley W, Delgado D (2017). Virtual reality for resuscitation using an iPhone. Resuscitation..

[42] Javaheri H, Gruenerbl A, Monger E, Gobbi M, Lukowicz P (2018). Stayin' Alive: An Interactive Augmented: Reality CPR Tutorial. Proceedings of the 2018 ACM International Joint Conference and 2018 International Symposium on Pervasive and Ubiquitous Computing and Wearable Computers.

[43] Kadosawa M, Makino M. An AR-based self-training system of chest compression as CPR. ProcSPIE. 2019;1104918.

[44] Kardong-Edgren S, Farra SL, Alinier G, Young HM. A call to unify definitions of virtual reality. Clinical Simulation in Nursing. 2019;31:28–34.

[45] Cheng A, Auerbach M, Calhoun A, Mackinnon R, Chang TP, Nadkarni V, et al. Building a community of practice for researchers: the International Network for Simulation-Based Pediatric Innovation, Research and Education. Simul Healthc. 2018;13(3S Suppl 1):S28–s34. 10.1097/SIH.0000000000000269 29117090

