# Supplementary material for: The use of virtual reality and augmented reality to enhance cardio-pulmonary resuscitation: a scoping review
Source: Adv Simul (Lond). 2021 Apr 12;6:11. doi: 10.1186/s41077-021-00158-0 (PMC8040758; doi:10.1186/s41077-021-00158-0)
Supplement: Supplementary file 1 — Additional file 1. The studies included in the scoping review. [file 41077_2021_158_MOESM1_ESM.docx]

# Additional file 1. The studies included in the scoping review

| **Title** | **Authors** | **Publication Year** | **Journal** |
| --- | --- | --- | --- |
| Virtual reality enhanced mannequin (VREM) that is well received by resuscitation experts | Semeraro F, Frisoli A, Bergamasco M, Cerchiari EL. | 2009 | Resuscitation |
| Immersive virtual reality simulations in nursing education | Kilmon CA, Brown L, Ghosh S, Mikitiuk A. | 2010 | Nursing education perspectives |
| Mini VREM Project | Semeraro F, Frisoli A, Bergamasco M, Cerchiari EL. | 2010 | Resuscitation |
| 3D immersive cardiopulmonary resuscitation (CPR) trainer | Tian Y, Raghuraman S, Yang Y, Guo X, Prabhakaran B. | 2014 | ACM conference proceedings |
| Collaborative virtual reality based advanced cardiac life support training simulator using virtual reality principle | Khanal P, Vankipuram A, Ashby A, et al. | 2014 | Journal of biomedical informatics |
| Design and development of a virtual reality simulator for advanced cardiac life support training | Vankipuram A, Khanal P, Ashby A, et al. | 2014 | IEEE Journal of biomedical an health informatics |
| Adapting guidelines for Google glass: the case of pediatric CPR. | Ehrler F, Siebert J, Haddad K, et a | 2016 | Studies in Health Technology and Informatics |
| Engineering student education in cardiopulmonary resuscitation via virtual reality. | Ohley W. | 2016 | Resuscitation |
| Development and evaluation of a corrective feedback system using augmented reality for the high-quality cardiopulmonary resuscitation training | Higashi E, Fukagawa K, Kasimura R, Kanamori Y, Minazuki A, Hayashi H | 2017 | IEEE Conference Proceedings |
| Randomised clinical simulation designed to evaluate the effect of telemedicine using Google Glass on cardiopulmonary resuscitation (CPR). | Pérez Alonso N, Pardo Rios M, Juguera Rodriguez L, et al. | 2017 | Emergency medicine journal |
| Virtual reality for resuscitation using an iPhone | Ohely W, and Delago D | 2017 | Resuscitation |
| Adherence to AHA guidelines when adapted for augmented reality glasses for assisted pediatric cardiopulmonary resuscitation: a randomized controlled trial | Siebert JN, Ehrler F, Gervaix A, et al. | 2017 | Journal of medical internet research |
| Stayin' Alive: an interactive augmented - reality CPR tutorial | Javaheri H, Gobbi M, Grünerbl A, Lukowicz P, and Monger E.. | 2018 | IEEE Conference Proceedings |
| Comparing a virtual reality mobile app vs. a standard mobile app for CPR training | Leary M, Almodovar A, Patel J, et al | 2018 | Circulation |
| Clinical instructors' perceptions of virtual reality in health professionals' cardiopulmonary resuscitation education | Wong M, Chue S, Jong M, Benny H, Zary N. | 2018 | SAGE open medicine |
| Virtual reality CPR: a new way to learn CPR | Semeraro F, Ristagno G, Giulini G, et al. | 2018 | Resuscitation |
| Development of CPR instruction by use of augmented reality on an I-phone. | Ohley W | 2018 | Resuscitation |
| Virtual reality cardiopulmonary resuscitation (CPR): Comparison with a standard CPR training mannequin | Semeraro F, Ristagno G, Giulini G, et al. | 2019 | Resuscitation |
| Successful virtual reality cardiopulmonary resuscitation training in schools: digitally linking a physical manikin to a virtual lifesaving scenario | Gent L, Sarno D, Coppock K, Axelrod D. | 2019 | Circulation |
| The effectiveness of augmented reality in infant education: a BLS and CPR learning study in 5 year-old students | Belmonte J, Sánchez S, Belmonte G. | 2019 | PIXEL-BIT- REVISTA DE MEDIOS Y EDUCACION |
| Comparing the effects on learning outcomes of tablet-based and virtual reality-based serious gaming modules for basic life support training: randomized trial | Aksoy E. | 2019 | JMIR Serious Games |
| Virtual reality simulation technology for cardiopulmonary resuscitation training: an innovative hybrid system with haptic feedback | Almousa O, Prates J, Yeslam N, et al. | 2019 | Simulation and gaming |
| CPR virtual reality training simulator for schools | Vaughan N, John N, Rees N. | 2019 | International conference on cyberworlds. |
| Towards an affordable virtual reality solution for cardiopulmonary resuscitation training | Liyanage S U, Jayaratne L, Wickramasinghe M, Munasingh A. | 2019 | International conference on cyberworlds. |
| An AR-based self-training system of chest compression as CPR | Kadosawa M, Makino M. | 2019 | Proceedings of SPIE |
| Using an immersive virtual reality system to assess lay provider response to an unannounced simulated sudden cardiac arrest in the out-of-hospital setting | Leary M, Almodovar A Jr, Buckler DG, Bhardwaj A, Blewer AL, Abella BS. | 2019 | Simulation healthcare |
| Back to reality: A new blended pilot course of basic life support with virtual reality | Semeraro F, Ristagno G, Giulini G, et al. | 2019 | Resuscitation |
| Performance monitoring via functional near infrared spectroscopy for virtual reality based basic life support training | Aksoy E, Izzetoglu K, Baysoy E, Agrali A, Kitapcioglu D, Onaral B. | 2019 | Frontiers in Neuroscience |
| Use of a virtual reality device for basic life support training: prototype testing and an exploration of users' views and experience | Bench S, Winter C, Francis G. | 2019 | Simulation in healthcare |
| Observing the stages of bystander intervention in virtual reality simulation | Buckler DG, Almodovar A, Snobelen P, Abella BS, Blewer A, Leary M. | 2019 | World Journal of Emergency medicine. |
| Virtual reality in cardiopulmonary resuscitation training: A randomized trial | Cerezo Espinosa C, Segura Melgarejo F, Melendreras Ruiz R, et al. | 2019 | Emergencias |
| Comparison of virtual reality versus standard video for point-of-training feedback after cardiopulmonary resuscitation simulation: a mixed methods study | Gillespie R, Nicholson J, Bickerdale S, Frith G, Hassan T. | 2019 | Resuscitation |
| A virtual reality feedback system for high quality CPR training | Gonzalez G. Pena M.S. Bergeron H.E. et al | 2019 | ASAIO journal |
| Comparing bystander response to a sudden cardiac arrest using a virtual reality CPR training mobile app versus a standard CPR training mobile app | Leary M, McGovern SK, Chaudhary Z, Patel J, Abella BS, Blewer AL. | 2019 | Resuscitation |
| Effectiveness of learning with flipped learning with augmented reality in school health education | Lopez-Belmonte, J. Pozo-Sanchez, S. Fuentes-Cabrera, A.; Romero-Rodriguez, J. M. | 2020 | Journal of sport and health research |
| Augmented reality learning environment for basic life support and defibrillation training: usability study. | Ingrassia PL, Mormando G, Giudici E, et al. | 2020 | Journal of medical internet research |
| Feasibility of an augmented reality cardiopulmonary resuscitation training system for health care providers | Balian S, McGovern SK, Abella BS, Blewer AL, Leary M. | 2020 | Circulation |
| Successful virtual reality cardiopulmonary resuscitation training in schools: Digitally linking a physical manikin to a virtual lifesaving scenario | Gent L, Sarno D, Coppock K, Axelrod D. | 2020 | Circulation |
| A comparison of CPR quality using an augmented reality application versus a standard audio-visual feedback manikin | McGovern S, Balian S, Bhardwaj A, Abella B, Blewer AL, Leary M. | 2020 | Circulation |
| Effect of face-to-face vs virtual reality training on cardiopulmonary resuscitation quality: A randomized clinical trial | Nas J, Thannhauser J, Vart P, et al. | 2020 | JAMA Cardiology |
| The use of immersive and virtual reality technologies to enable nursing students to experience scenario-based, basic life support training- Exploring the impact on confidence and skills. | Rushton MA, Drumm IA, Campion SP, OʼHare JJ. Rushton et al | 2020 | Computer informatics nursing |
