# Supplementary material for: The use of virtual reality and augmented reality to enhance cardio-pulmonary resuscitation: a scoping review
Source: Adv Simul (Lond). 2021 Apr 12;6:11. doi: 10.1186/s41077-021-00158-0 (PMC8040758; doi:10.1186/s41077-021-00158-0)
Supplement: Supplementary file 2 — Additional file 2. The impact factor of the journals publishing VR AR CPR studies, in this review. [file 41077_2021_158_MOESM2_ESM.docx]

# Additional file 2. The impact factor of the journals publishing VR AR CPR studies, in this review

| **Source Title** | **Impact Factor*** |
| --- | --- |
| Circulation | 18.881 |
| JAMA Cardiology | 11.866 |
| Resuscitation | 5.863 |
| Journal of Medical Internet Research | 4.671 |
| Frontiers in Neuroscience | 3.648 |
| Emergencias | 3.608 |
| JMIR Serious Games | 3.531 |
| ASAIO Journal | 2.678 |
| Emergency Medicine Journal | 2.491 |
| Simulation in Healthcare | 1.761 |
| World Journal of Emergency Medicine | 1.743 |
| Computers Informatics Nursing | 1.321 |
| Studies in Health Technology and Informatics | 0.44 |
| Journal of sport and health research | not listed |
| SAGE Open Med | not listed |
| Journal of Biomedical Informatics | not listed |
| Simulation and Gaming | not listed |
| Nursing Education Perspectives | not listed |

* Impact factor of original publishing journals used.
